# Infectious SIV resides in adipose tissue and induces metabolic defects in chronically infected rhesus macaques

**DOI:** 10.1186/s12977-016-0260-2

**Published:** 2016-04-27

**Authors:** Jacob Couturier, Neeti Agarwal, Pramod N. Nehete, Wallace B. Baze, Michael A. Barry, K. Jagannadha Sastry, Ashok Balasubramanyam, Dorothy E. Lewis

**Affiliations:** Division of Infectious Diseases, Department of Internal Medicine, The University of Texas Health Science Center at Houston, Houston, TX 77030 USA; Graduate School of Biomedical Sciences, The University of Texas Health Science Center at Houston, Houston, TX USA; Division of Diabetes, Endocrinology and Metabolism, Department of Medicine, Diabetes Research Center, Baylor College of Medicine, Houston, TX USA; Department of Veterinary Sciences, The University of Texas MD Anderson Cancer Center, Bastrop, TX USA; Department of Molecular Medicine, Mayo Clinic, Rochester, MN USA; Department of Immunology, The University of Texas MD Anderson Cancer Center, Houston, TX USA; Endocrine Service, Ben Taub General Hospital, Houston, TX USA

**Keywords:** Adipose tissue, CD4 T cells, HIV latency, HIV reservoirs, Rhesus macaques, SIV reservoirs

## Abstract

**Background:**

HIV reservoirs pose major challenges to viral eradication. The main cellular reservoirs include CD4 T cells and macrophages, whereas anatomic reservoirs are thought to be primarily lymphoid tissues. Adipose tissue represents a potentially important non-lymphoid location for HIV replication and persistence because the stromal-vascular-fraction (AT-SVF) contains activated innate and adaptive immune cells that increase in number during infections, obesity, and chronic inflammation.

**Results:**

Adipose tissue from two groups of SHIV-SF162p3-infected (~4 weeks acute infection) or SIVmac251-infected (~38 weeks chronic infection) rhesus macaques (N = 8 for each group) were studied for immune cell content, viral infectiousness, and metabolic health. The AT-SVF cells from SHIV-infected monkeys contained abundant memory CD4 and CD8 T cells, with fewer NKT cells and macrophages, and no B cells. Proviral DNA (Gag and Env) was readily detectable by nested PCR in AT-SVF cells from multiple adipose depots (subcutaneous and visceral) of acutely infected monkeys, but mostly from visceral fat. More importantly, viral outgrowth assays using input CD4 T cells derived from AT-SVF cells or peripheral blood of chronically infected monkeys resulted in robust replication of infectious virus from both AT-SVF and peripheral blood CD4 T cells. Chronically infected monkeys also experienced adipocyte dysfunction (suppression of major adipogenic genes) and systemic dyslipidemia (decreased serum total cholesterol and free fatty acids, and increased triglycerides), similar to metabolic abnormalities of HIV patients.

**Conclusions:**

Adipose tissues of SIV-infected rhesus macaques become major compartments for infected immune cells, which in turn induce defects in adipose tissue metabolism.

**Electronic supplementary material:**

The online version of this article (doi:10.1186/s12977-016-0260-2) contains supplementary material, which is available to authorized users.

## Background

Eradication and immune control of HIV is difficult due to the establishment of reservoirs and anatomic sanctuaries [1, 2]. Memory CD4 T cells and macrophages are the primary hosts and cellular reservoirs for HIV in humans and SIV in non-human primates. Anatomic reservoirs for infected immune cells include lymphoid (lymph nodes, spleen, thymus, bone marrow, and GALT) and non-lymphoid (lungs, skin, liver, kidneys, reproductive, and nervous system) tissues. Antiretroviral therapy (ART) reduces the systemic viral load below the detection limits of clinical assays in most patients, but viral replication typically resumes if ART is interrupted [3]. ART is also incompletely effective in certain locations, such as CNS tissue and lymph nodes, due to unique cellular barriers to ART drugs [4, 5]. Hence, understanding cellular and anatomic reservoirs for HIV, and how they may prevent adequate tissue penetration by ART drugs, is critical to achieving a cure.

A major organ that has been overlooked hitherto for a role in HIV pathogenesis is adipose tissue. It is well-known that a range of immune cells reside in or migrate into adipose tissues and affect their metabolic signals [6, 7], but adipocytes and other adipose tissue-resident cells interact with CD4 T cells and macrophages which has important implications for HIV pathogenesis. Anatomically, adipose tissue predominantly underlies the skin (subcutaneous fat) and surrounds abdominal organs (visceral fat). However, adipocytes are also intimately associated with most lymphoid tissues—lymph nodes are tightly encapsulated by adipose tissue, adipocytes are abundant within bone marrow, and the thymus gradually becomes filled with adipocytes during adult aging. Such an intricate arrangement is important during infections or immunological reactions because adipocytes are major sources of energy and survival signals for immune cells [8]. At the cellular level, adipose tissue is heterogeneous and composed of mature adipocytes (the predominant fraction containing lipid droplets and triglycerides), and the stromal-vascular-fraction (AT-SVF) which includes mainly preadipocytes (adipocyte precursors) and fibroblasts, mesenchymal stem cells (MSC), endothelial cells, and immune cells. Extensive studies in humans and mice have demonstrated the presence of virtually every type of innate and adaptive leukocyte within adipose tissue during normal conditions, the composition and functions of which change dramatically in response to disease and inflammation. Importantly for HIV infection, the adipose tissue-resident CD4 T cells, which are predominantly activated memory CD4 T cells (CD45RO+CD69+HLA.DR+CD25+) [9–13], phenotypically resemble those in other tissues where HIV persists. We recently demonstrated for the first time the presence of memory CD4 T cells and HIV proviral DNA within the stromal-vascular-fraction of virally-suppressed ART-treated patients [12]. In addition, we showed by in vitro co-culture experiments that primary human adipocytes enhance HIV replication in CD4 T cells [12]. As adipocytes are ubiquitous endocrine cells that extensively regulate immunity and disease, these findings warrant further investigation into the role of adipose tissue in HIV replication and persistence.

SIV-infected rhesus macaques remain the best animal model for HIV infection and viral pathogenesis. In the present study, samples of adipose tissue were acquired from rhesus macaques at necropsy, which were infected with SHIV-SF162p3 for ~4 weeks (N = 8), or SIVmac251 for ~38 weeks (N = 8), and not treated with antiretroviral drugs. We hypothesized that memory CD4 T cells in adipose tissue harbors infectious virus, and that these untreated infected monkeys would develop metabolic complications similar to HIV-infected humans. Although the original purpose of these infected monkeys did not include the study of adipose tissue, examination of their fat tissue demonstrated that infiltration of adipose tissue by CD4 T cells infected with infectious virus is a regular event during SIV infection. Long-term infection of monkeys also resulted in some metabolic abnormalities resembling those of HIV patients. The present findings highlight the prevalence and stability of the viral reservoir in adipose tissue, and provide novel evidence for viral-induced metabolic dysfunction.

## Results

### Establishment of reservoirs of memory CD4 T cells and SIV throughout adipose tissue during primary infection

Viral eradication is challenged by the rapidity with which SIV spreads throughout lymphoid tissues (within 7 days), and stable SIV reservoirs established (within 3 days) following infection of rhesus macaques [14, 15]. Adipose tissue inflammation and dysfunction typically involves the accumulation and regulatory activities of numerous innate and adaptive immune cells, particularly proinflammatory memory T cells, macrophages, and NKT cells. Thus, it is plausible that primary infection also includes viral dissemination and establishment of reservoirs in adipose tissue as adipocytes are intricately associated with most lymphoid tissues.

**Fig. 1:**
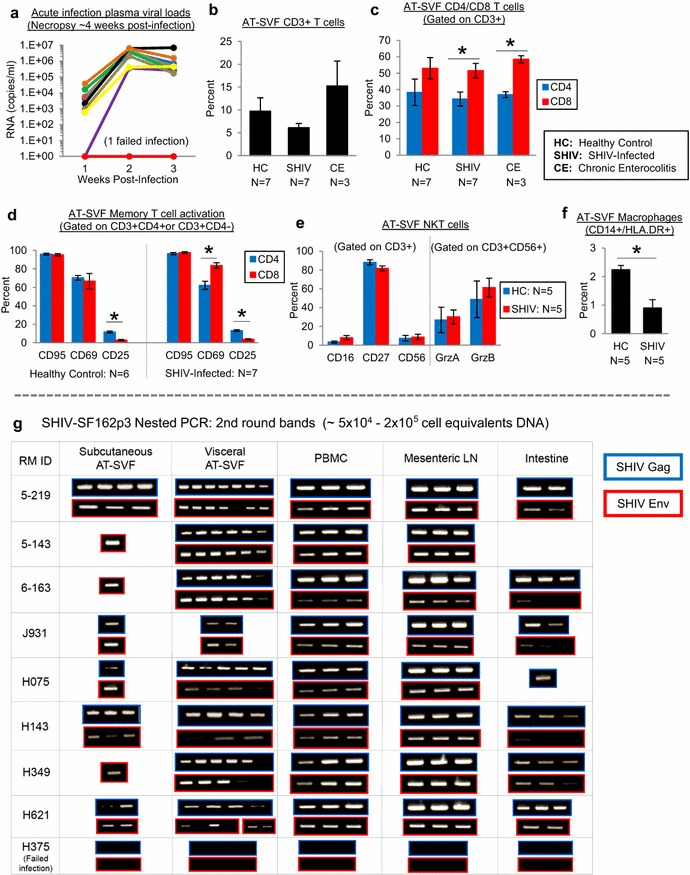
Leukocyte and proviral distribution in the adipose tissue stromal-vascular-fraction (AT-SVF) of acutely infected monkeys. Visceral and subcutaneous adipose tissue samples were harvested from rhesus macaques at necropsy. AT-SVF cells were isolated as described in “Methods” section and examined for leukocyte content (T cells, NKT cells, and macrophages) by flow cytometry, and SHIV DNA detection (Gag and Env) by nested PCR. **a** Plasma viral loads of SHIV-SF162p3-infected rhesus macaques (infection was successful for eight out of nine monkeys). **b**, **c** Mean ± SEM percentages of AT-SVF CD3+, or CD4 (CD3+CD4+) and CD8 (CD3+CD4−) T cells of uninfected healthy and SHIV-infected monkeys (*p < 0.05). For additional comparisons, AT-SVF T cells of uninfected rhesus macaques with chronic enterocolitis were also examined. **d**–**f ** Mean ± SEM percentages of AT-SVF T cell memory (CD95) and activation (CD25 and CD69), NKT cell (CD16, CD27, CD56, GrzA, and GrzB), and macrophage (CD14 and HLA.DR) markers of uninfected healthy or SHIV-infected monkeys (*p < 0.05). **g** Nested PCR detection (2nd round gel bands) of SHIV-SF162p3 Gag and Env genes in DNA extracted from subcutaneous and visceral AT-SVF cells (~5 × 10^4^–2 × 10^5^ cell equivalents of DNA) of infected monkeys. DNA from PBMC, mesenteric lymph nodes, and intestinal tissue were also examined for comparison. PCR replicates of 6–9 were tested for AT-SVF cells, and replicates of three were tested for PBMC, MLN, and intestinal tissues

To examine the leukocyte and proviral distribution in adipose tissue during primary infection, adipose tissue samples of acutely infected (SHIV-SF162p3) rhesus macaques were first studied. Figure 1a shows plasma viral loads after intra-rectal infection of nine monkeys, in which infection was unsuccessful for one monkey. Monkeys were necropsied ~4 weeks post-infection, and ~5–15 g of adipose tissue samples harvested from abdominal subcutaneous and visceral regions. AT-SVF cells were isolated from adipose tissue as described in “Methods” section, and visceral AT-SVF cells examined for activated memory T cells (CD3, CD4, CD8, CD95, CD25, and CD69), NKT cells (CD3, CD16, CD27, CD56, GrzA, and GrzB), B cells (CD19 and CD80), and macrophages (CD14 and HLA.DR) by flow cytometry (Additional file 1 shows the general method of AT-SVF isolation and gating schemes for flow cytometry analyses). As mentioned in “Methods” section, over the course of the present study, adipose tissue samples were also obtained from various uninfected rhesus macaques that were healthy or experiencing health complications such as chronic enterocolitis, and were studied for comparison to SHIV-infected monkeys.

From total AT-SVF cells, CD3+ T cells accounted for ~9.8 % for uninfected healthy monkeys, ~6.1 % for SHIV-infected monkeys, and ~15.3 % for uninfected monkeys with chronic enterocolitis (Fig. 1b). Additionally, whereas CD8 and CD4 T cell levels were similar in AT-SVF of uninfected healthy monkeys (ratio of 1.38, *p* = 0.18), the ratio of AT-SVF CD8 to CD4 T cells increased during infection and disease (1.51 during SHIV infection, and 1.59 during chronic enterocolitis, *p* < 0.05) (Fig. 1c). For both uninfected healthy and SHIV-infected monkeys, nearly all of the AT-SVF CD4 and CD8 T cells were memory T cells (>94 % CD95+), which expressed tissue-resident and activation markers (62–84 % CD69+ and 3–13 % CD25+) (Fig. 1d). However, CD4 T cells expressed more CD25 (12–13 %) compared to CD8 T cells (3–4 %, *p* < 0.0001), possibly indicative of Treg subsets. AT-SVF cells of uninfected healthy and SHIV-infected monkeys were also comprised of similar levels of NKT cells (Fig. 1e), which were 3–8 % CD16+, 82–88 % CD27+, and 7–9 % CD56+ (gated on CD3+ cells), and 27–30 % GrzA+ and 49–61 % GrzB+ (gated on CD3+/CD56+ cells). Lastly, CD3-/CD14+/HLA.DR+ macrophages represented a small proportion (<1–2 %) of total AT-SVF cells of uninfected healthy and SHIV-infected monkeys (Fig. 1f), and CD3-/CD19+/CD80+ B cells were not present in AT-SVF cells (<0.8 %, data not shown). Thus, primary SIV infection is associated with homing of the major reservoir cell, memory CD4 T cells, into adipose tissue, which is further associated with minor changes of T cell and macrophage distributions.

To examine the SHIV-SF162p3 proviral content in AT-SVF cells of acutely infected monkeys, a nested PCR assay was utilized since AT-SVF cell numbers were limited and CD4 T cells comprised a small proportion (usually less than ~5 %) of total AT-SVF cells. Approximately 5 × 10^4^–2 × 10^5^ subcutaneous and visceral AT-SVF cell equivalents of DNA were used for each nested PCR reaction (6–9 replicates for each sample). For comparison, DNA extracted from other tissues including PBMC, mesenteric lymph nodes, and intestinal tissues were also examined (replicates of three for each sample). SHIV DNA (Gag and Env) was readily detectable in AT-SVF samples of all eight infected monkeys (but absent in tissues of the failed infection monkey), with more consistent detection in visceral AT-SVF samples compared to subcutaneous AT-SVF (Fig. 1g). Additionally, the 2nd round PCR bands of AT-SVF samples were gel-purified and sequenced, which showed that Gag and Env sequences were virtually identical amongst all monkeys (Additional file 2), consistent with previous reports demonstrating high sequence homology and minimal viral evolution in tissues during early infection periods of rhesus macaques [16, 17]. These data show that memory CD4 T cells and SIV reservoirs are widely distributed throughout adipose tissue of rhesus macaques during primary infection.

### Infectious SIV harbored by adipose tissue CD4 T cells

Despite the efficacy of antiretroviral therapy to suppress viral replication in HIV patients, persistence of latently-infected CD4 T cells that harbor dormant, but replication-competent, provirus remains a major obstacle to eradication. These infected cells are unaffected by ART drugs and undetectable by antiviral CD8 T cells, and viral replication typically resumes within a month if therapy is discontinued. Whereas tissues such as lymph nodes containing CD4 T cells are important sources of rebound viremia, the contribution of other tissues such as the GALT is less clear [3, 18]. However, the presence of infectious virus in adipose tissue depots could also contribute to systemic viremia.

**Fig. 2:**
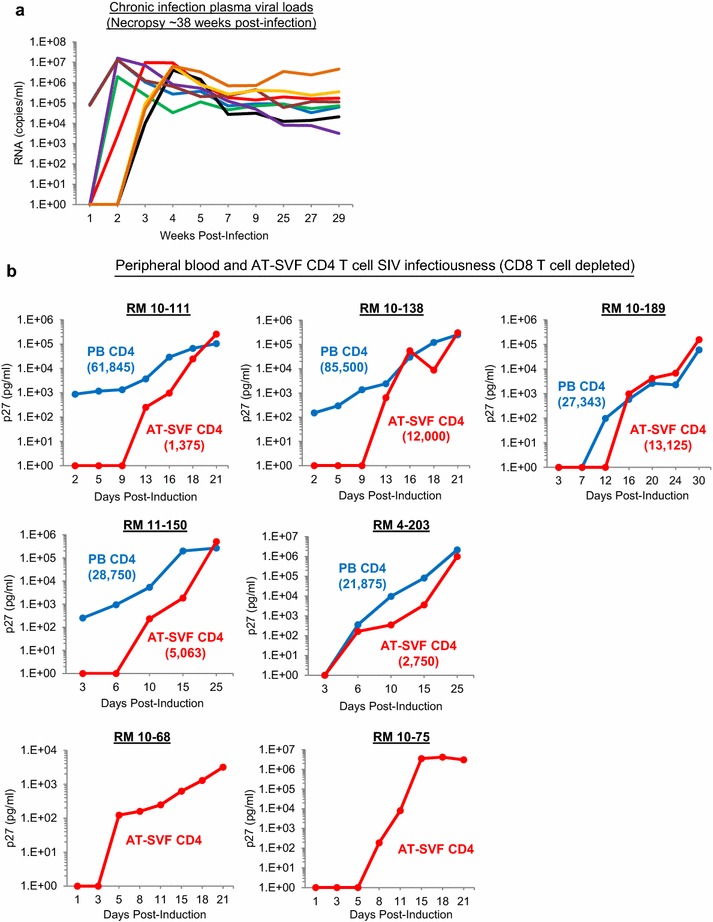
Infectiousness of SIV in peripheral blood and AT-SVF CD4 T cells of chronically infected rhesus macaques. **a** Plasma viral loads of chronically SIVmac251-infected monkeys. **b** Viral outgrowth of SIV from peripheral blood (PB) and adipose tissue (AT-SVF) CD4 T cells. PBMC was isolated from peripheral blood and AT-SVF cells isolated from adipose tissue of infected monkeys at necropsy. CD4 T cells were then purified from PBMC or AT-SVF cells, and activated with PHA + IL-2 and co-cultured with M8166 cells for 3–4 weeks in viral outgrowth assays as described in “Methods” section. Graphs show extracellular p27 levels from PBMC- or AT-SVF-derived CD4 T cells for five infected monkeys (numbers in parentheses indicate the input cell number at the start of the assay). For two infected monkeys (RM 10–68 and 10–75, bottom two plots), peripheral blood was unavailable, and an exact AT-SVF CD4 T cell count undetermined (but estimated at ~5 × 10^3^–2 × 10^4^ cells)

To assess the infectiousness of SIV-infected CD4 T cells in adipose tissue, we conducted viral outgrowth assays on CD4 T cells purified from the AT-SVF of chronically SIVmac251-infected monkeys (infected for ~38 weeks, Fig. 2a shows plasma viral loads). CD4 T cells purified from peripheral blood or AT-SVF cells were serially diluted (twofold) six times and activated with PHA+IL-2 for 2 days, followed by addition of M8166 cells for propagation of induced SIV (measured by extracellular p27). For five infected monkeys (RM’s 10–111, 10–138, 10–189, 11–150, and 4–203), viral induction from peripheral blood CD4 T cells was examined in parallel to AT-SVF CD4 T cells, and for two infected monkeys (RM’s 10–68 and 10–75), peripheral blood was unavailable and viral induction from only AT-SVF CD4 T cells was examined. For simplicity, Fig. 2b shows viral replication levels by the lowest input cell number in which extracellular p27 was detectable within 2 weeks post-induction (viral replication levels for all dilutions are shown in Additional file 3). In all seven monkeys examined, infectious SIV was inducible from AT-SVF CD4 T cells. Based on five monkeys, infectiousness appeared to be mostly comparable between peripheral blood and AT-SVF CD4 T cells by 3–4 weeks of culture, despite the starting input number of AT-SVF CD4 T cells (~1.4 × 10^3^–1.3 × 10^4^) being substantially less than peripheral blood CD4 T cells (~2.2 × 10^4^–8.6 × 10^4^) as shown in Fig. 2b.

We additionally assessed if infectious virions may be sequestered or trapped by mature adipocytes in the adipocyte fraction of isolated adipose tissue samples, as it has been suggested that HIV may non-productively infect adipocytes [19]. From three infected monkeys, ~6 × 10^6^ M8166 cells were incubated with rotation at 37 °C in 5–8 ml of visceral floater fraction suspension from each monkey for 8 h, followed by centrifugation, washing, and removal of dead M8166 cells by density-gradient centrifugation. 3 × 10^6^ M8166 cells were then cultured for up to 3 weeks and extracellular p27 measured, in which p27 was not detected (data not shown), indicating a lack of infectious virion sequestration or trapping by adipocytes.

**Fig. 3:**
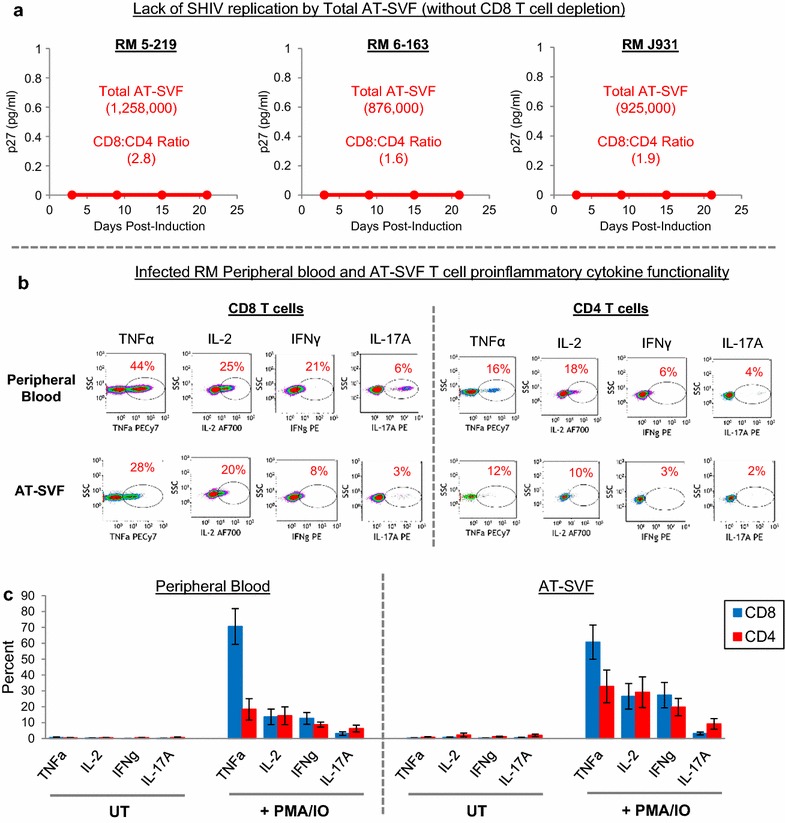
Multi-functionality of CD8 T cells in adipose tissue of infected rhesus macaques. **a** Lack of viral outgrowth from *total* AT-SVF cells (without CD8 T cell depletion) of three acutely SHIV-SF162p3-infected monkeys. Shown are input numbers of total AT-SVF cells at the start of the assay, and the ratio of AT-SVF CD8 to CD4 T cells. **b**, **c** Proinflammatory cytokine functionality of peripheral blood and AT-SVF T cells of chronically infected monkeys. Isolated PBMC or AT-SVF cells of SIVmac251-infected monkeys were untreated (UT) or stimulated with PMA/IO (in the presence of brefeldin) for 5 h. Cells were then stained for CD3, CD8, TNFa, IL-2, IFNγ, and IL-17A and analyzed by flow cytometry. Shown are representative cytokine dotplots gated on peripheral blood or AT-SVF CD3+/CD8+ or CD3+/CD8− T cells after PMA/IO activation, and mean ± SEM cytokine expression (N = 4–5)

To further assess the function of adipose tissue CD8 T cells, viral outgrowth assays were attempted using *total* AT-SVF cells (without CD8 depletion) of three SHIV-infected monkeys. Approximately 8.8 × 10^5^–1.3 × 10^6^ starting input total AT-SVF cells were activated with PHA+IL-2, then co-cultured with M8166 cells for up to 3 weeks. However, SHIV induction was not observed (Fig. 3a), possibly due to the viral suppressive function of CD8 T cells as the majority of AT-SVF CD3+T cells were CD8+(AT-SVF CD8:CD4 ratios of 1.6–2.8). Additionally, the peripheral blood and visceral AT-SVF CD8 and CD4 T cells of 4–5 SIV-infected monkeys were examined for proinflammatory cytokine functionality using flow cytometry ICS assays (Fig. 3b). Cytokine phenotypes of AT-SVF T cells were ~61 % TNFα+, ~27 % IL-2+, ~27 % IFNγ+, and ~3 % IL-17A+ for CD8 T cells, and ~33 % TNFα+, ~29 % IL-2+, ~20 % IFNγ+, and ~9 % IL-17A+ for CD4 T cells, which were similar to peripheral blood T cell cytokine profiles, suggesting that adipose tissue CD8 T cells are highly functional. Thus, CD4 T cells in adipose tissue of SIV-infected rhesus macaques are infected with replication-competent and infectious virus, but such viral inducibility does not occur in the presence of adipose tissue CD8 T cells.

### Induction of metabolic perturbations by SIV infection in the absence of antiretroviral drugs

Metabolic dysfunction (such as dyslipidemias, hyperlipolysis, and decreased leptin and adiponectin production) and adipocyte abnormalities (such as differentiation block due to blunted expression of key adipogenic transcription factors) are prevalent during HIV infection. Whereas some of these defects have been attributed to the adverse effects of ART drugs, similar complications also occur in untreated or ART-naïve HIV patients. Additionally, viral proteins such as Vpr, Nef, and Tat impair adipocyte functions directly [20–24].

**Fig. 4:**
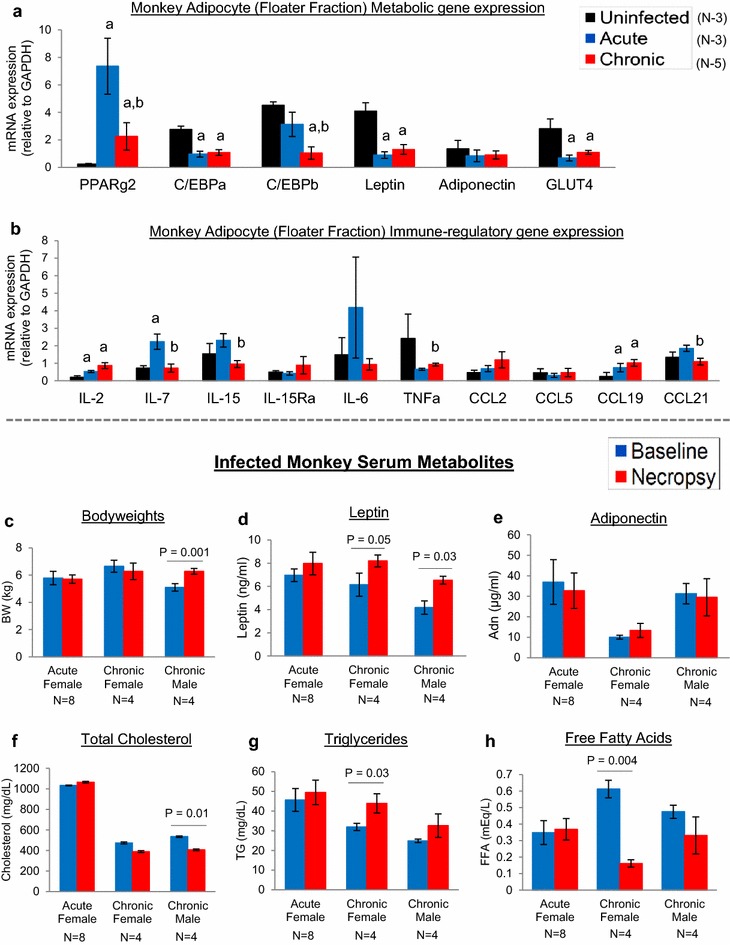
Alterations of adipocyte homeostasis and systemic metabolism during SIV infection of rhesus macaques. **a**, **b** mRNA expression of major adipogenic and immunological factors by visceral adipocytes of uninfected healthy (N = 3), acutely infected (N = 3) and chronically infected (N = 5) monkeys at necropsy. Adipose tissue samples were digested with collagenase, then washed and centrifuged as described in “Methods” section to harvest mature adipocytes (floater fraction). mRNA was extracted from adipocytes and gene expression measured by real-time PCR (**a** indicates *p* < 0.05 compared to uninfected monkeys, and **b** indicates *p* < 0.05 comparing chronic versus acute infection monkeys). **c**–**h** Mean ± SEM bodyweights and serum total cholesterol, triglycerides, free fatty acids, leptin, and adiponectin of acutely infected (eight females) and chronically infected (four females and four males) monkeys at baseline (prior to infection) and at necropsy

To determine if SIV infection induces adipose metabolic defects in monkeys, we examined visceral adipocyte mRNA expression of C/EBPα, C/EBPβ, PPARγ2, leptin, adiponectin, and GLUT4, as well as serum total cholesterol, lipids (triglycerides and free fatty acids), leptin, and adiponectin. As adipocytes extensively interact with T cells, we also examined adipocyte expression of factors that regulate T cell stimulation, survival, and migration (IL-2, IL-7, IL-15/IL-15Rα, IL-6, TNFα, CCL2, CCL5, CCL19, and CCL21). For adipocyte mRNA analyses, visceral adipose tissue was acquired from three uninfected healthy monkeys for comparison to three acutely infected and five chronically infected monkeys. Compared to uninfected monkeys, differential expression of PPARγ2, C/EBPα, C/EBPβ, leptin, and GLUT4 was observed by adipocytes of infected monkeys (Fig. 4a). Relative to uninfected monkeys, PPARγ2 expression was increased 30.2-fold for acutely infected and 9.3-fold for chronically infected monkeys, whereas C/EBPα was decreased 2.9-fold for acutely infected and 2.5-fold for chronically infected monkeys, C/EBPβ was decreased 4.3-fold for chronically infected monkeys, leptin was decreased 4.5-fold for acutely infected and 3.1-fold for chronically infected monkeys, and GLUT4 was decreased 4.1-fold for acutely infected and 2.6-fold for chronically infected monkeys (*p* < 0.05). Adiponectin expression by adipocytes was similar between uninfected and infected monkeys. In conjunction with dysregulated expression of adipogenic factors, adipocytes of uninfected and infected monkeys also expressed important immune-regulatory factors (Fig. 4b). Adipocyte expression of IL-2, IL-7, and CCL19 was increased 1.6 to 3-fold in infected monkeys compared to uninfected monkeys (*p* < 0.05), whereas expression of other cytokines and chemokines were similar between uninfected and infected monkeys, suggesting that adipocytes express factors that may contribute to the homing and survival of infected CD4 T cells in adipose tissue.

Consistent with the dysregulated expression of adipocyte factors, abnormal serum levels of important metabolic factors (total cholesterol, triglycerides, free fatty acids, leptin, and adiponectin) were observed in infected monkeys. When grouped by gender (eight acutely infected females, and four each chronically infected males and females) (since gender differences are well-known to influence metabolic parameters), and compared between baseline and necropsy time points, more changes of serum metabolites were observed in chronically infected monkeys. Bodyweights of acutely and chronically infected female monkeys were unchanged, but modestly increased for chronically infected males (Fig. 4c). Serum leptin levels were unchanged during acute infection, but modestly increased during chronic infection (Fig. 4d), whereas serum adiponectin levels were unchanged during acute and chronic infection (Fig. 4e). Serum total cholesterol was unchanged during acute infection, but decreased for chronically infected males (Fig. 4f). Serum triglycerides and free fatty acids were unchanged during acute infection, whereas triglycerides were increased and free fatty acids decreased for chronically infected females (Fig. 4g, h). Lastly, these alterations of adipocyte and serum metabolism by chronically infected monkeys were observed in conjunction with high systemic viral loads (Fig. 2a), increased peripheral blood CD8 T cells, and increased serum inflammatory cytokines (Additional file 4), although changes in other tissues affected by HIV infection likely impact metabolic homeostasis as well. These data suggest that adipose tissue and systemic metabolic homeostasis of rhesus macaques are disrupted by SIV infection, which resemble metabolic complications of HIV patients.

## Discussion

A more complete understanding of cellular and anatomic compartments for HIV is important to permit targeted efforts to eradicate latent HIV infection. The present study demonstrates that adipose tissue of SIV-infected rhesus macaques is a major site for memory CD4 T cells harboring infectious virus. In light of the extensive characterization of adipose tissue-immune system interactions in recent years, it is significant that this study demonstrated an abundance of infectious virus in adipose tissue. Adipocytes express numerous chemokines that recruit memory CD4 T cells and monocytes into adipose tissue, where T cells and macrophages differentiate into proinflammatory subsets during disease and inflammation. Additionally, adipose tissue macrophages, stromal-vascular-fraction cells, and mature adipocytes activate CD4 T cells via soluble factors and direct contact mechanisms, and furthermore promote T cell survival [25–27]. HIV replication by adipose tissue CD4 T cells and macrophages may also be regulated by TLR stimulation, as breaches of intestinal epithelia result in infiltration of microbial products into visceral fat [28]. Furthermore, microbial pathogens such as *M. tuberculosis* can establish reservoirs in adipocytes [29], which may influence the pathogenesis of HIV co-infections in adipose tissue. Future investigations should focus on better understanding the mechanisms of antiviral immunity and efficacy of antiretroviral therapies in adipose tissue.

The rhesus macaques in the present study were infected with two different strains of virus (SHIV-SF162p3 or SIVmac251) for different time periods (~4 or ~38 weeks), and represented a range of viral loads, immunological parameters (CD4/CD8 cell counts and systemic cytokine levels), ages (2-16yo), and gender. Despite this biological range, notable was the ease and consistency in which memory CD4 T cells, SHIV provirus, and infectious SIV could be detected in the adipose tissue of all infected monkeys studied (Figs. 1, 2). This corroborates recent studies demonstrating HIV- or SIV-infected immune cells in both subcutaneous and visceral fat of infected humans and cynomolgus macaques examined [12, 13], further emphasizing the regularity of viral dissemination throughout adipose tissue. In these investigations, it has been essential to examine specifically the stromal-vascular-fraction of adipose tissue (following collagenase digestion to separate these cells from the mature adipocyte fraction), as mature adipocytes comprise the majority of adipose tissue, and only less than 5–10 % of total AT-SVF cells were CD4 T cells or macrophages. As shown in Fig. 1, the accumulation of infected immune cells in adipose tissue during acute infection was accompanied by minor changes in T cell and macrophage distribution, possibly due to the short time period of infection. Compared to uninfected healthy monkeys, the adipose tissue CD4:CD8 T cell ratio of infected monkeys was slightly increased (suggesting increased CD4 depletion or increased CD8 homing), whereas macrophages were decreased and NKT cells unchanged (although examination of additional M1/M2 macrophage markers, and NKT cell markers such as NKG2A may indicate more distinct changes of these subsets). These findings indicate that adipose tissue, particularly visceral fat, become a reservoir for SIV early during infection, and remain an infectious viral reservoir during longer-term infections.

The infectiousness of adipose tissue CD4 T cell SIV was mostly comparable to peripheral blood virus (Fig. 2), and such replication-competence suggests that adipose tissue infected CD4 T cells may contribute to systemic viremia or viral rebound. SIV replication was inducible from adipose tissue CD4 T cells of all seven chronically infected monkeys examined, and from as few as ~1 × 10^4^ input AT-SVF CD4 T cells from most monkeys. This finding is also consistent with the report by Damouche et al., which demonstrated higher replication-competence by adipose tissue CD4 T cells compared to peripheral blood CD4 T cells in ART-treated HIV patients [13]. This may be because CD4 T cells in adipose tissue, as well as in other extravascular tissues, are predominantly memory CD4 T cells and the most productive host for HIV, by contrast to peripheral blood which contains mostly resting memory CD4 T cells and more naïve T cells. However, in the presence of multi-functional proinflammatory CD8 T cells, which comprised the majority of CD3 T cells in adipose tissue, propagation of infectious virus was inhibited (Fig. 3). Additionally, NKT cell populations (CD3+/CD16+/CD27+/CD56+/GrzA+/GrzB+) were also observed in AT-SVF of infected monkeys (Fig. 1e), which may exert additional antiviral functions. The proinflammatory cytokine phenotypes of AT-SVF T cells of infected monkeys are also consistent with a range of models of adipose tissue inflammation which collectively depict that adipose tissue of healthy and lean humans and mice contain predominantly anti-inflammatory Tregs and Th2 cells, but shifts toward proinflammatory Th1 and Th17 cells during obesity and other diseases [10, 30–34]. Such changes in CD4 T cell differentiation may yield more susceptible targets for HIV, as Th1 and Th17 cells are more productive hosts for HIV compared to Tregs or Th2 cells [35–37]. It is unclear how viral replication levels by CD4 T cells and macrophages in adipose tissue compare to other tissues, and how well antiviral immunity suppresses replication, but the large mass of adipose tissue in the body suggests that it may be a large infectious reservoir.

Adipose tissue dysfunction is common amongst HIV patients, manifested by a range of clinical effects from dyslipidemia to lipoatrophy and fat “redistribution” [38, 39]. The etiology of these manifestations is multifactorial, including the adverse effects of ART drugs, direct effects of viral proteins such as Vpr and Nef, and indirect effects of inflammation secondary to immune activation within adipose depots [40, 41]. The present study of untreated acutely and chronically infected monkeys suggests that more metabolic changes occur as infection progresses over time. Additionally, interactions amongst activated immune cells and adipocytes in adipose tissue may promote inflammation that precedes the systemic metabolic changes. Whereas acutely infected monkeys mostly experienced metabolic stability, chronically infected monkeys experienced reduction of serum total cholesterol and increased triglycerides (Fig. 4f–h). Serum leptin was also increased in chronically infected monkeys (Fig. 4d), which for the males is consistent with their modest weight gain (Fig. 4c). This weight change may also be related to the increased densities of adipocytes and SVF cells observed in chronically SIV-infected (15 months) cynomolgus macaques [13]. Leptin can also promote T cell survival [42], which could influence HIV persistence in adipose tissue. Quantification of gene expression of factors critical for adipocyte differentiation and function (PPARγ2, C/EBPα, C/EBPβ, leptin, adiponectin, and GLUT4) indicated that SIV infection decreased the expression of most of these genes (Fig. 4a), which may increase the risk for fat redistribution, lipoatrophy, or insulin resistance. Although metabolic changes in chronically infected monkeys occurred in conjunction with increased plasma cytokines (Additional file 4), factors derived from other tissues affected by viral infection, particularly lymphoid tissues proximal to adipose tissue, likely contribute to adipocyte dysfunction as well.

Some of these metabolic changes parallel adipose metabolic defects demonstrated in other animal models in the absence of ART drugs. For example, mouse models have shown that circulating Vpr induces dyslipidemias, increases energy expenditure, and suppresses adipogenic gene expression and adipocyte growth in vitro [20, 22]. Soluble Nef can also mediate dyslipidemias in SIV-infected rhesus macaques (infection for 2 months following 6 months of a proatherogenic diet of high cholesterol and saturated fats) by decreasing liver ABCA1 expression and impairing reverse cholesterol transport [21]. These findings are further supported by in vitro studies demonstrating more directly the negative effects of Vpr, Nef, and Tat on adipocyte functions [22, 23, 43, 44]. Thus, in the absence of antiretroviral treatments, the metabolic changes in the infected monkeys of the present study provides further evidence that viral infection within the stromal-vascular-fraction of adipose depots and the resultant immunological alterations can perturb metabolism and adipose tissue homeostasis.

Immune-regulatory factors that promote T cell homing and HIV replication was also expressed by adipocytes (Fig. 4b). Expression of these factors has been previously demonstrated in human and murine adipocytes [45–47], but not in adipocytes of non-human primates. The common gamma-chain cytokines IL-2, IL-7, and IL-15 are major regulators of T cell survival and homeostatic stimulation, but in combination with proinflammatory cytokines such as IL-6 and TNFα, and with other adipocyte-secreted factors, can upregulate T cell activation and HIV replication [12, 48, 49]. Although these factors are generally upregulated in adipose tissue during infections and disease, substantial increases of immune cells were not observed in adipose tissue of acutely infected monkeys compared to uninfected monkeys as shown in Fig. 1, possibly due to the short time period of infection or the requirement for additional chemotactic signals from other stromal-vascular-fraction cells. Consistent with previous reports showing relatively high expression of CCL19 compared to other chemokines in human and murine adipocytes [50], we observed higher expression of CCL19 by adipocytes of infected monkeys compared to uninfected monkeys. During HIV infection, CCL19 and CCL21 enhance steps of viral post-integration latency in CD4 T cells [51], which in combination with other stimulatory agents in adipose tissue, may facilitate the complete replication cycle of HIV. Thus, adipocyte immunokines may contribute to the homing and survival of infected CD4 T cells and macrophages in adipose tissue, thus “seeding” the viral reservoir in this organ.

It is possible that the antiviral efficacy of ART drugs may be compromised in adipose tissue, since some of these drugs are lipophilic and can be sequestered within the relatively larger-sized adipocytes [52, 53]. Such sequestration might prevent the drugs from penetrating the stromal-vascular compartment harboring infected immune cells. In virally-suppressed ART-treated HIV patients, low-level viral replication in lymphoid tissue sanctuary sites, in association with inadequate penetration by ART drugs, has recently been reported as an important mechanism of viral persistence [4, 5]. Such sanctuary sites may exist in other tissues, and ongoing studies are investigating the penetration and efficacy of ART drugs in adipose tissue.

## Conclusions

A better understanding of HIV reservoirs and anatomic sanctuaries is essential for treatment and eradication efforts. SIV-infected rhesus macaques represent the best animal model for HIV pathogenesis, and in the present study, fat tissues of infected monkeys are demonstrated to contain abundant memory CD4 T cells, highly infectious virus, and proinflammatory immune cells. SIV infection furthermore induced metabolic complications in the absence of antiretroviral drugs, some of which resembled metabolic conditions of HIV patients. Thus, this major endocrine organ represents an expansive location for SIV and HIV reservoirs that gradually becomes dysfunctional during viral infection.

## Methods

### Animals and infections

All animal experiments were approved by the Institutional Animal Care and Use Committee at the University of Texas MD Anderson Cancer Center and were carried out according to the provisions of the Animal Welfare Act, PHS Animal Welfare Policy, and the principles of the NIH Guide for the Care and Use of Laboratory Animals, and the policies and procedures of the University of Texas MD Anderson Cancer Center. Rhesus macaques (*Macaca mulatta*) of Indian origin were maintained in the specific pathogen-free breeding colony at the Michael Keeling Center for Comparative Medicine and Research of The University of Texas MD Anderson Cancer Center (Bastrop, Texas). The chamber size for the animals was 44′W × 88′H × 160′D. Monkeys were given water ad libitum, and fed a commercial monkey diet (Harlan). Additional enrichment was provided in the form of manipulanda, visual stimulation or auditory stimulation, and combinations thereof. Animals were monitored daily, including weekends and holidays. Anesthetics/analgesics were used to minimize any discomfort, distress, pain, and injury the animal might experience. Animals were euthanized with ketamine (11 mg/kg), followed by Beuthanasia (1 ml/10 lbs). If any animal was moribund, unresponsive to treatment, could not eat or drink, was severely sick, or had symptoms of SAIDS, it was euthanized as per guidelines. Animals were anesthetized during procedures to minimize discomfort.

For infections, monkeys were fasted for a minimum of 24 h prior to exposure. Monkeys were first anesthetized with 10 mg/kg of body weight ketamine intramuscularly and 0.5 mg/kg xylazine, then placed in a sternal position with the pelvis propped at 45°. Monkeys were infected by intra-rectal inoculation of 1000 TCID_50_ clonal stocks of SHIV-SF162p3 or SIVmac251 (NIH AIDS Reagent Program). The infected monkey was then returned to its cage and kept tilted at 45° until full recovery from anesthesia. RNA plasma viral loads were measured as previously described [54]. The SHIV-SF162p3-infected monkeys were necropsied ~4 weeks post-infection, and SIVmac251-infected monkeys necropsied ~38 weeks post-infection, and are referred to as *acutely* or *chronically* infected monkeys, respectively, throughout the manuscript.

During the course of the study, adipose tissue samples were also acquired from rhesus macaques that were uninfected and mostly healthy, or uninfected but afflicted with chronic enterocolitis. These samples were utilized for comparisons to the infected monkeys (i.e. adipose tissue immune cell distribution or adipocyte gene expression studies) as indicated in the Results section. The demographic details of all the rhesus macaques used in the study are shown in Additional file 5.

### Isolation of stromal-vascular-fraction (AT-SVF) cells from adipose tissue, and PBMC isolation from peripheral blood

During necropsy of monkeys, adipose tissue samples (~5–15 g) were harvested from abdominal subcutaneous and visceral regions and immediately processed for AT-SVF isolation. For AT-SVF isolation, ~1–3 g of adipose tissue at a time was minced with scissors, then digested with 1 mg/ml collagenase type II (Sigma) in 6 ml PBS in 15 ml conical tubes for 30–60 min (with rotation at 37 °C). The digest was then centrifuged to pellet the AT-SVF cells, whereas mature adipocytes (floater fraction) remained suspended due to lipid droplet buoyancy. The adipocytes were harvested and stored at  −80 °C for real-time PCR analyses. The AT-SVF cells were then washed with PBS/2 % FBS, filtered through 70 µm mesh, and maintained in complete RPMI-1640 medium (10 % FBS, 2 mM l-glutamine, 0.1 mM MEM nonessential amino acids, 2 mM sodium pyruvate, 25 mM HEPES, and 1X antibiotic–antimycotic) at 37 °C/5 % CO_2_ prior to experiments. AT-SVF cell yields were usually ~2 × 10^5^–8 × 10^5^ cells per gram of adipose tissue.

PBMC were isolated from EDTA-anticoagulated whole blood by overlay onto Ficoll-Paque PLUS (GE Healthcare) and density-gradient centrifugation. PBMC were then washed and maintained in complete RPMI medium prior to experiments.

### Flow cytometry

Antibodies for flow cytometry analyses were obtained from Biolegend, BD Biosciences, or eBioscience. For examination of PBMC and AT-SVF leukocytes, cells were washed with PBS/2 % FBS and stained with the following mabs: CD3-Pacblue, CD4-PerCPCy5.5, CD8-FITC, CD95-PE, CD25-PECy7, and CD69-APC for T cells; CD3-Pacblue, CD16-AF700, CD56-PE, GrzB-AF647, and either GrzA-PerCPCy5.5 or CD27-PerCPCy5.5 for NKT cells (intracellular GrzA and GrzB were stained using BD Biosciences Cytofix/Cytoperm solutions); CD19-PE and CD80-FITC for B cells; and CD14-Pacblue, CD16-PE, and HLA.DR-APCCy7 for macrophages. Isotype controls were used to assess non-specific binding, and compensation settings determined with single-color and fluorescence-minus-one (FMO) controls. Data was acquired with a Gallios Flow Cytometer and analyzed with Kaluza software (Beckman-Coulter).

For intracellular cytokine secretion (ICS) assays, PBMC or AT-SVF cells were stimulated with PMA/IO in the presence of GolgiPlug (BD Biosciences Leukocyte Activation Cocktail) for 5 h. Cells were washed with PBS/2 % FBS and stained with viability dye and surface mabs (CD3-PerCPCy5.5, CD8-FITC or CD8-APCCy7) for 30 min, followed by fixation/permeabilization with Cytofix/Cytoperm solution (BD Biosciences) for 30 min. Cells were then washed with Perm/Wash buffer and incubated with intracellular mabs (TNFα-PECy7, IL-2-AF700, IFNγ-PE, or IL-17A-PE) for 30 min. Cells were washed and analyzed by flow cytometry.

### Nested PCR and DNA sequencing

DNA extraction was conducted using QIAamp DNA Micro Kit (QIAGEN). Nested PCR was performed for detection of SHIV-SF162p3 DNA (SIVmac239 Gag and HIV-1 Env) in cells of acutely infected monkeys, and primers are listed in Additional file 6. 1st round reactions included 40 μl PCR SuperMix (Life Technologies), 200–300 nM primers, 0.1 μg DNA, and 10 μl water; PCR conditions were one cycle at 94 °C (2 min), then 35–40 cycles at 94 °C (30 s), 55 °C (30 s), and 72 °C (55 s), then final extension at 72 °C (10 min). 2nd round reactions were 40 μl PCR SuperMix, 200–300 nM primers, 3 μ1 of 1st round product, and 10 μl water, and PCR conditions were one cycle at 94 °C (2 min), then 35–40 cycles at 94 °C (30 s), 55 °C (30 s), and 72 °C (30 s), then final extension at 72 °C (10 min). 2nd round PCR products were then gel-purified and sequenced by the Baylor College of Medicine DNA Sequencing Core (using ABI 3130XL Genetic Analyzer and BigDye Terminator). Multiple sequence alignments were conducted with Clustal Omega.

### CD4 T cell purification and viral outgrowth assays

CD4 T cells were purified from PBMC or AT-SVF cells of infected rhesus macaques using bead-based positive selection kits (Stemcell Technologies), and purity assessed by flow cytometry. Induction and propagation of SIV replication was conducted using a previously described viral outgrowth method with minor modifications [55]. Cells were serially diluted (twofold) six times and activated with 5 µg/ml PHA-L+50 ng/ml IL-2 in complete RPMI medium (3 ml) for 2 days. Medium was then replenished and PHA-L removed, followed by addition of 2 × 10^5^ M8166 cells (NIH AIDS Reagent Program) for viral propagation. Cells were cultured for up to 3–4 weeks, in which medium was replenished every 3–4 days and cultures split every 6–7 days. Extracellular p27 was measured by sandwich ELISA kits (XpressBio).

### Serum analyte measurements

Serum of infected monkeys was collected by centrifugation of anti-coagulated whole blood and storage at −80 °C. Serum cytokines were measured using the MILLIPLEX Non-Human Primate Immunology Multiplex Assay (EMD Millipore), and analyzed with a Bio-Plex 200 System (Bio-Rad). Serum total cholesterol was measured by Infinity Cholesterol Liquid Stable Reagent (Thermo Scientific), serum triglycerides were measured using Triglycerides Reagent kit (Thermo Scientific), and serum free fatty acids were measured using NEFA-HR(2) kit (Wako Diagnostics). Serum leptin was measured using Non-Human Primate Leptin competitive ELISA kit (NeoScientific), and serum adiponectin (Acrp30) was measured using LEGEND MAX sandwich ELISA kit (Biolegend).

### Real-time PCR

Total RNA was extracted from the floater fraction adipocytes of collagenase-digested adipose tissue samples using RNeasy Lipid Tissue Mini Kit (QIAGEN). RNA was reverse-transcribed to cDNA, and SYBR Green real-time PCR performed using the ABI 7300 System (Applied Biosystems). Primers (Additional file 7) were designed using Primer Express and synthesized by Sigma Genosys. Reaction conditions were one cycle at 95 °C (10 min), followed by 40 cycles of 95 °C (30 s), 55 °C (60 s), and 72 °C (60 s), and ended with one cycle of 95 °C (60 s), 55 °C (30 s), and 95 °C (30 s). GAPDH was used as the calibrator for normalization of gene expression, and fold changes were calculated using 2^−ΔΔCT^ formula.

### Statistics

Data were analyzed with GraphPad Prism or Microsoft Excel. Comparisons utilized two-tailed student’s *t* test (paired or unpaired as appropriate), and *p* values less than 0.05 were considered significant.

## Additional files

10.1186/s12977-016-0260-2 General method for isolation of stromal-vascular-fraction (AT-SVF) cells from adipose tissue of rhesus macaques, and subsequent analyses. (A) 30-60 mins collagenase digestion of solid adipose tissue samples from rhesus macaques is followed by washing and centrifugation, allowing for separation of mature adipocytes (floater fraction) from the stromal-vascular-fraction (AT-SVF) cells. AT-SVF cells were then analyzed by flow cytometry, nested PCR, and viral outgrowth assays, and floater fraction adipocytes analyzed for mRNA expression. (B) Sample flow cytometry gating schemes for examination of AT-SVF T cells, NKT cells, macrophages, and B cells.
10.1186/s12977-016-0260-2 Sequence confirmation of nested PCR products, and lack of viral diversity in AT-SVF of acutely infected rhesus macaques. PCR products from SHIV Gag (A) and Env (B) 2nd round nested PCR reactions of subcutaneous and visceral AT-SVF DNA of eight infected monkeys (shown in Figure 2G) were gel-purified, sequenced, and aligned with Clustal-Omega software. Yellow-highlighted nucleotides indicate a nucleotide difference compared to other nucleotides in the alignment column (sequences include nucleotide positions A:1667 to G:2085 relative to SIVmac239 Gag, and G:7042 to C:7329 relative to HIV-1 HXB2 Env, indicated in red).
10.1186/s12977-016-0260-2 Infectiousness of SIV in peripheral blood and AT-SVF CD4 T cells of chronically infected rhesus macaques. PBMC was isolated from peripheral blood and AT-SVF cells isolated from adipose tissue of infected monkeys at necropsy. CD4 T cells were then purified from PBMC or AT-SVF cells, serially diluted (twofold) six times, then activated with PHA+IL-2 and co-cultured with M8166 cells for 3-4 weeks in viral outgrowth assays as described in Methods. Graphs show log extracellular p27 levels from peripheral blood- or AT-SVF-derived CD4 T cells (numbers in parentheses indicate the input cell number at the start of the assay).
10.1186/s12977-016-0260-2 Peripheral blood T cell counts and serum cytokine levels of acutely and chronically infected rhesus macaques. (A) Peripheral blood CD4 and CD8 T cell counts of infected monkeys at baseline (prior to infection) and at necropsy. (B-C) Serum cytokine levels of acutely and chronically infected monkeys at baseline and necropsy (*p<0.05, N=7-8 for each cytokine).
10.1186/s12977-016-0260-2 Demographics of infected and uninfected rhesus macaques used in the study.
10.1186/s12977-016-0260-2 Primers used for nested PCR detection of SHIV-SF162p3 Gag and Env genes in AT-SVF cells of acutely infected rhesus macaques.
10.1186/s12977-016-0260-2 Primers used for SYBR Green real-time PCR analyses of mature adipocytes (floater fraction) of rhesus macaques.
