# Supplementary material for: Infectious SIV resides in adipose tissue and induces metabolic defects in chronically infected rhesus macaques
Source: Retrovirology. 2016 Apr 27;13:30. doi: 10.1186/s12977-016-0260-2 (PMC4847269; doi:10.1186/s12977-016-0260-2)
Supplement: Supplementary file 1 — 10.1186/s12977-016-0260-2 General method for isolation of stromal-vascular-fraction (AT-SVF) cells from adipose tissue of rhesus macaques, and subsequent analyses. (A) 30-60 mins collagenase digestion of solid adipose tissue samples from rhesus macaques is followed by washing and centrifugation, allowing for separation of mature adipocytes (floater fraction) from the stromal-vascular-fraction (AT-SVF) cells. AT-SVF cells were then analyzed by flow cytometry, nested PCR, and viral outgrowth assays, and floater fraction adipocytes analyzed for mRNA expression. (B) Sample flow cytometry gating schemes for examination of AT-SVF T cells, NKT cells, macrophages, and B cells. [file 12977_2016_260_MOESM1_ESM.ppt]

## Slide 1
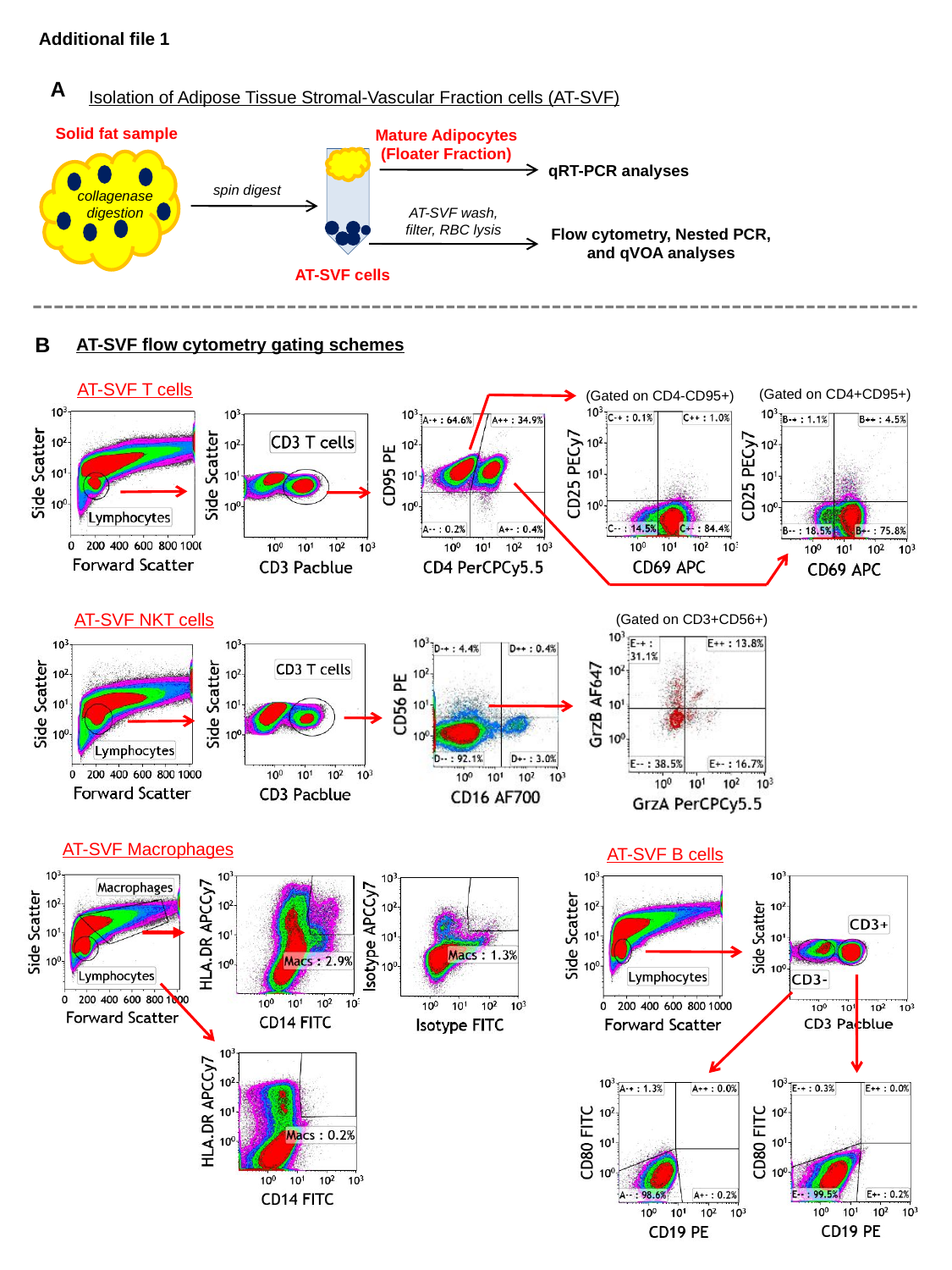

Additional file 1
A
Isolation of Adipose Tissue Stromal-Vascular Fraction cells (AT-SVF)
Solid fat sample
Mature Adipocytes
(Floater Fraction)
qRT-PCR analyses
spin digest
collagenase
digestion
AT-SVF wash,
filter, RBC lysis
Flow cytometry, Nested PCR,
and qVOA analyses
AT-SVF cells
B
AT-SVF flow cytometry gating schemes
AT-SVF T cells
(Gated on CD4+CD95+)
(Gated on CD4-CD95+)
AT-SVF NKT cells
(Gated on CD3+CD56+)
AT-SVF Macrophages
AT-SVF B cells
