# Supplementary material for: Infectious SIV resides in adipose tissue and induces metabolic defects in chronically infected rhesus macaques
Source: Retrovirology. 2016 Apr 27;13:30. doi: 10.1186/s12977-016-0260-2 (PMC4847269; doi:10.1186/s12977-016-0260-2)
Supplement: Supplementary file 2 — 10.1186/s12977-016-0260-2 Sequence confirmation of nested PCR products, and lack of viral diversity in AT-SVF of acutely infected rhesus macaques. PCR products from SHIV Gag (A) and Env (B) 2nd round nested PCR reactions of subcutaneous and visceral AT-SVF DNA of eight infected monkeys (shown in Figure 2G) were gel-purified, sequenced, and aligned with Clustal-Omega software. Yellow-highlighted nucleotides indicate a nucleotide difference compared to other nucleotides in the alignment column (sequences include nucleotide positions A:1667 to G:2085 relative to SIVmac239 Gag, and G:7042 to C:7329 relative to HIV-1 HXB2 Env, indicated in red). [file 12977_2016_260_MOESM2_ESM.ppt]

## Slide 1
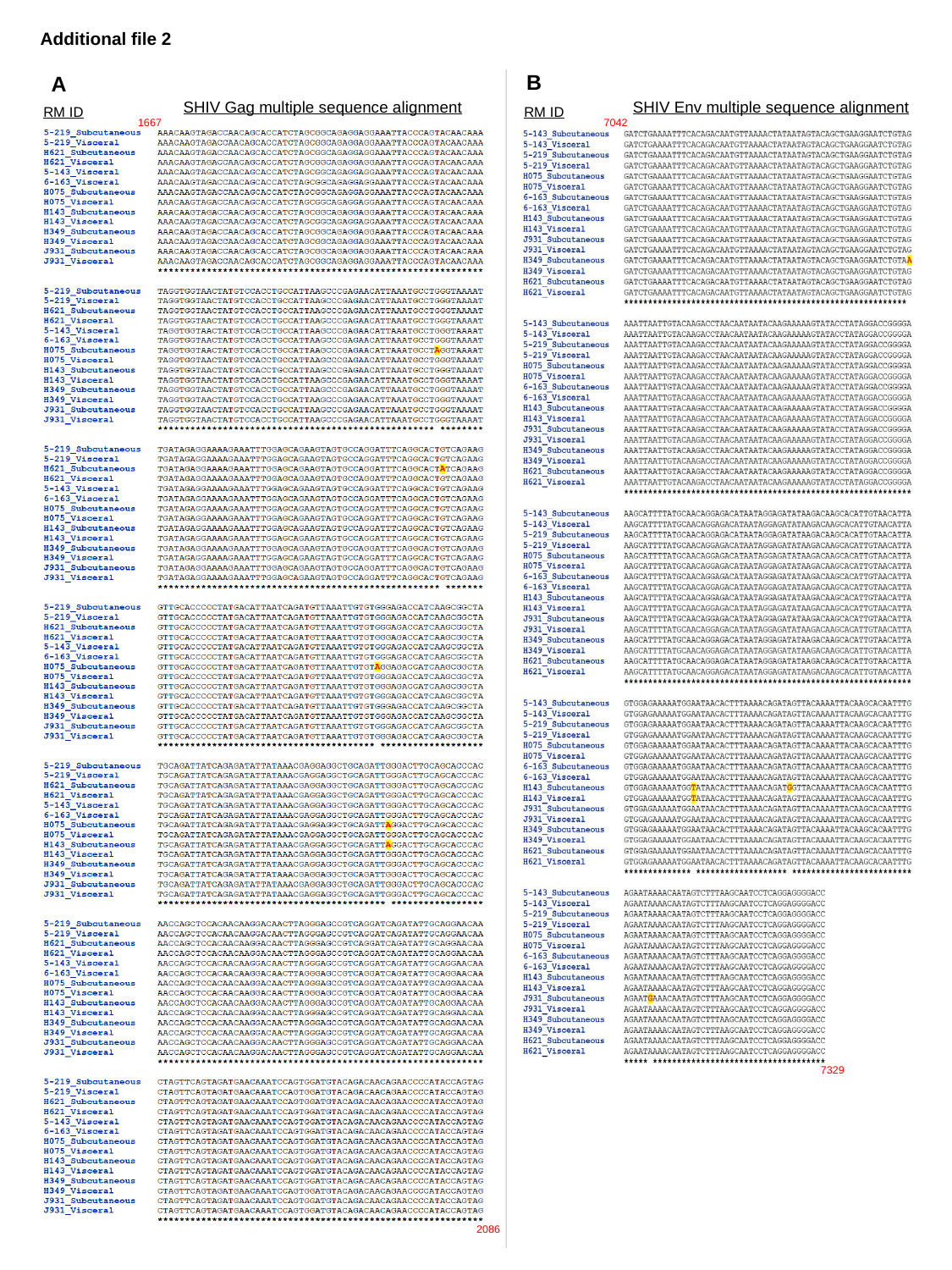

Additional file 2
B
A
SHIV Gag multiple sequence alignment
SHIV Env multiple sequence alignment
RM ID
RM ID
1667
7042
7329
2086
