# Supplementary material for: Infectious SIV resides in adipose tissue and induces metabolic defects in chronically infected rhesus macaques
Source: Retrovirology. 2016 Apr 27;13:30. doi: 10.1186/s12977-016-0260-2 (PMC4847269; doi:10.1186/s12977-016-0260-2)
Supplement: Supplementary file 3 — 10.1186/s12977-016-0260-2 Infectiousness of SIV in peripheral blood and AT-SVF CD4 T cells of chronically infected rhesus macaques. PBMC was isolated from peripheral blood and AT-SVF cells isolated from adipose tissue of infected monkeys at necropsy. CD4 T cells were then purified from PBMC or AT-SVF cells, serially diluted (twofold) six times, then activated with PHA+IL-2 and co-cultured with M8166 cells for 3-4 weeks in viral outgrowth assays as described in Methods. Graphs show log extracellular p27 levels from peripheral blood- or AT-SVF-derived CD4 T cells (numbers in parentheses indicate the input cell number at the start of the assay). [file 12977_2016_260_MOESM3_ESM.ppt]

## Slide 1
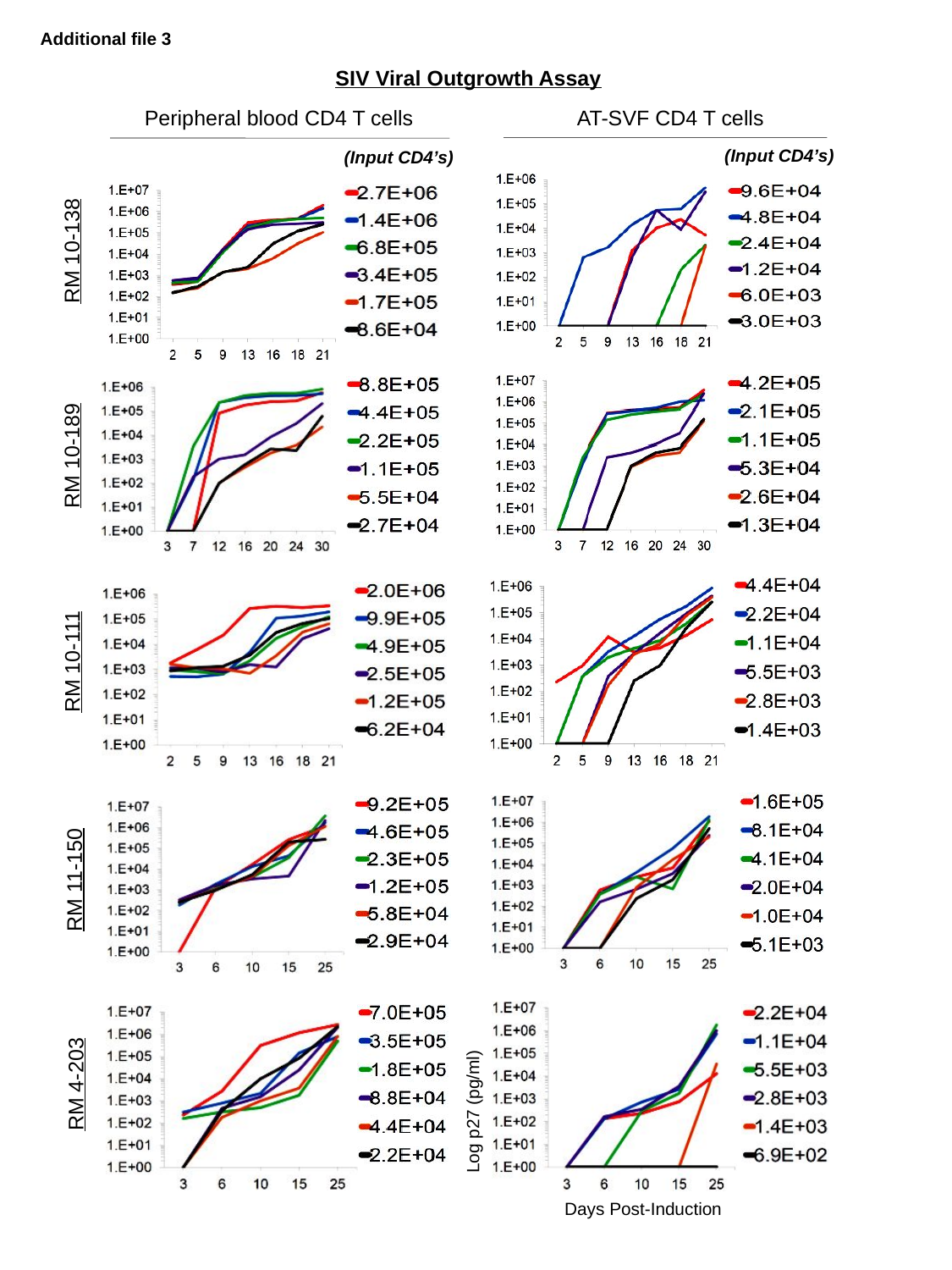

Additional file 3
SIV Viral Outgrowth Assay
Peripheral blood CD4 T cells
AT-SVF CD4 T cells
(Input CD4’s)
(Input CD4’s)
RM 10-138
RM 10-189
RM 10-111
RM 11-150
RM 4-203
Log p27 (pg/ml)
Days Post-Induction
