# Supplementary material for: Infectious SIV resides in adipose tissue and induces metabolic defects in chronically infected rhesus macaques
Source: Retrovirology. 2016 Apr 27;13:30. doi: 10.1186/s12977-016-0260-2 (PMC4847269; doi:10.1186/s12977-016-0260-2)
Supplement: Supplementary file 4 — 10.1186/s12977-016-0260-2 Peripheral blood T cell counts and serum cytokine levels of acutely and chronically infected rhesus macaques. (A) Peripheral blood CD4 and CD8 T cell counts of infected monkeys at baseline (prior to infection) and at necropsy. (B-C) Serum cytokine levels of acutely and chronically infected monkeys at baseline and necropsy (*p<0.05, N=7-8 for each cytokine). [file 12977_2016_260_MOESM4_ESM.ppt]

## Slide 1
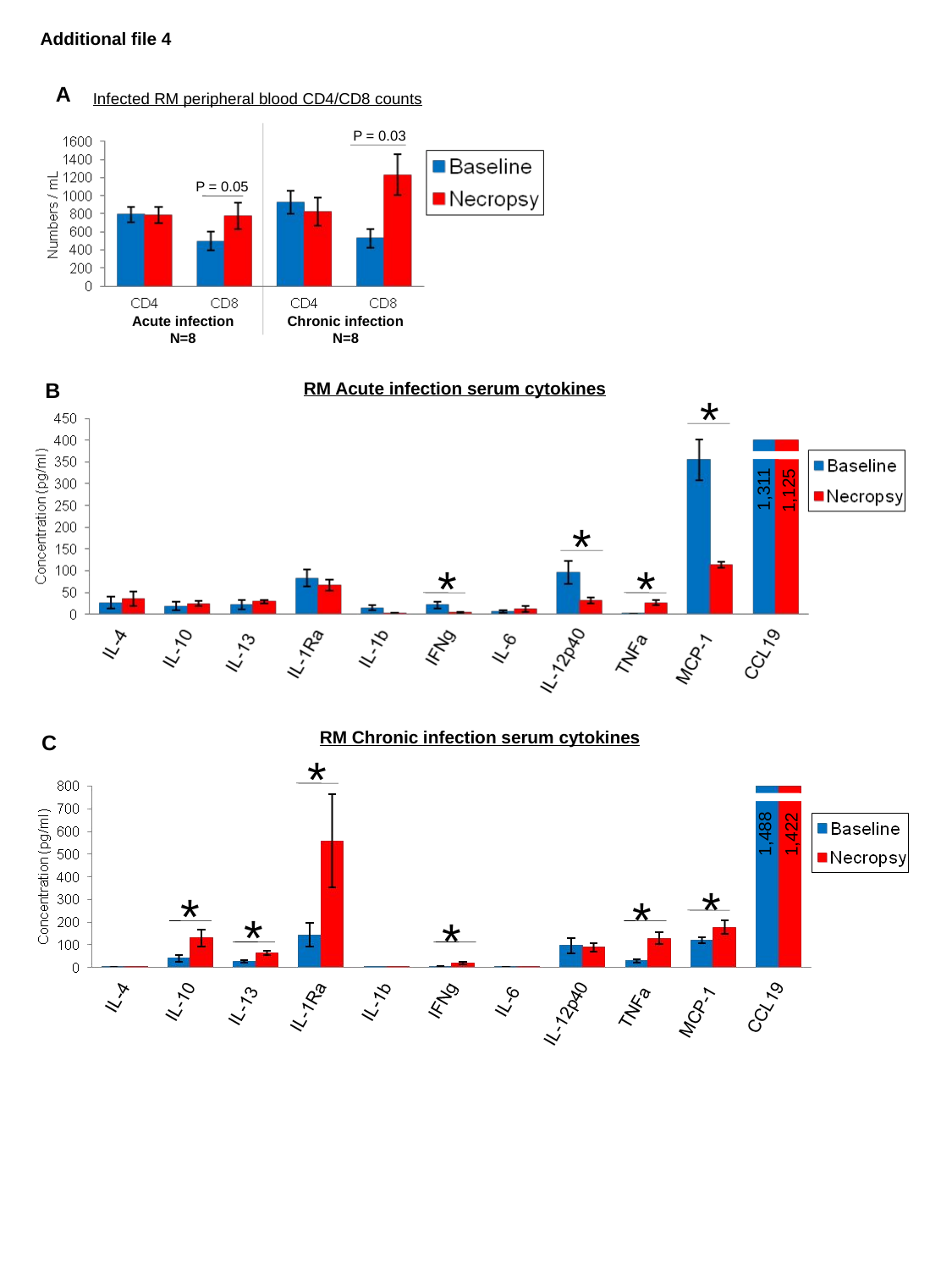

Additional file 4
A
Infected RM peripheral blood CD4/CD8 counts
P = 0.03
P = 0.05
Acute infection
N=8
Chronic infection
N=8
B
RM Acute infection serum cytokines
*
1,311
1,125
*
*
*
RM Chronic infection serum cytokines
C
*
1,488
1,422
*
*
*
*
*
