# Supplementary material for: Infectious SIV resides in adipose tissue and induces metabolic defects in chronically infected rhesus macaques
Source: Retrovirology. 2016 Apr 27;13:30. doi: 10.1186/s12977-016-0260-2 (PMC4847269; doi:10.1186/s12977-016-0260-2)
Supplement: Supplementary file 5 — 10.1186/s12977-016-0260-2 Demographics of infected and uninfected rhesus macaques used in the study. [file 12977_2016_260_MOESM5_ESM.ppt]

## Slide 1
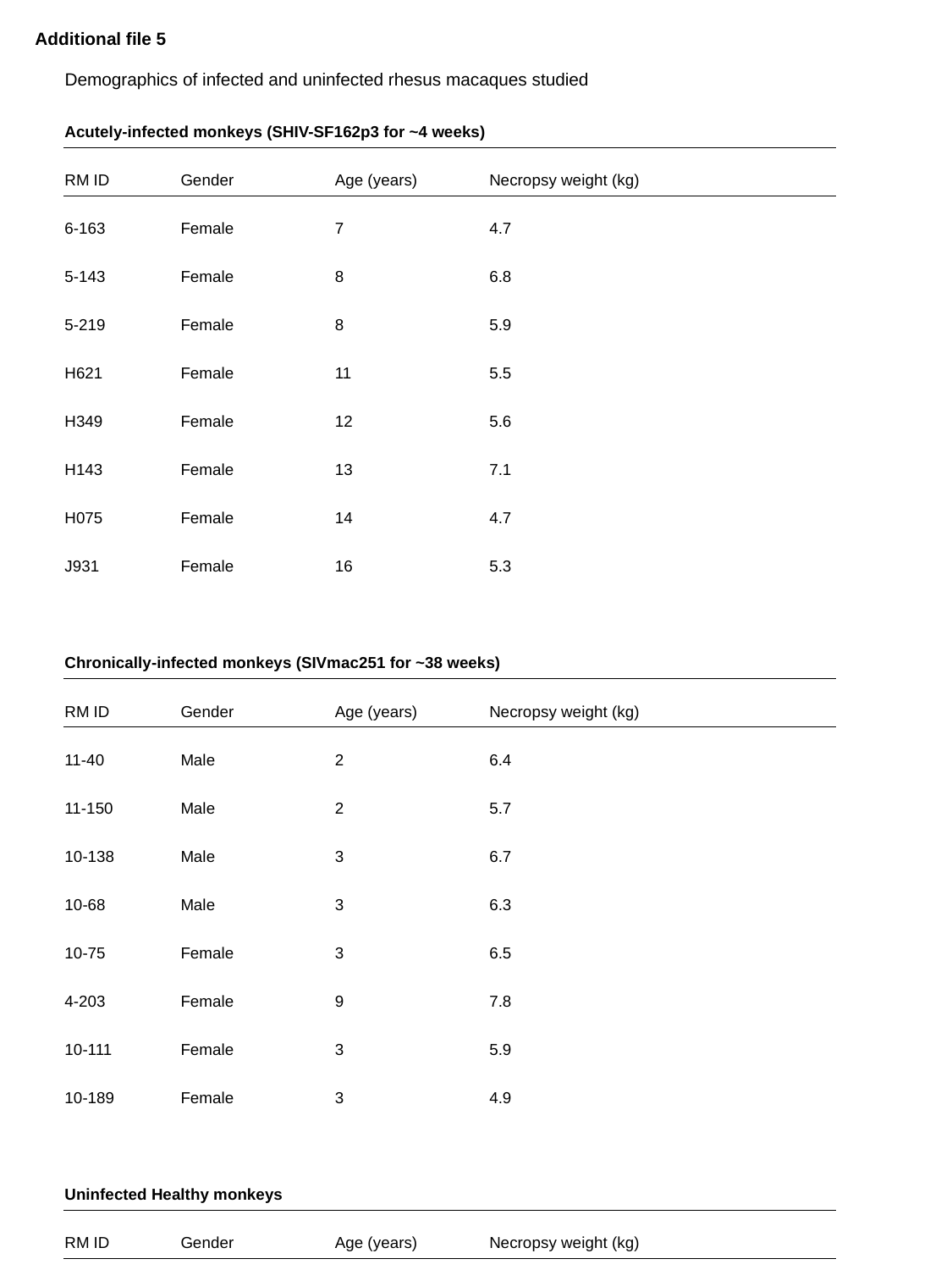

Additional file 5
Demographics of infected and uninfected rhesus macaques studied
| Acutely-infected monkeys (SHIV-SF162p3 for ~4 weeks) | | | | |
| --- | --- | --- | --- | --- |
| RM ID | Gender | Age (years) | Necropsy weight (kg) | |
| 6-163 | Female | 7 | 4.7 | |
| 5-143 | Female | 8 | 6.8 | |
| 5-219 | Female | 8 | 5.9 | |
| H621 | Female | 11 | 5.5 | |
| H349 | Female | 12 | 5.6 | |
| H143 | Female | 13 | 7.1 | |
| H075 | Female | 14 | 4.7 | |
| J931 | Female | 16 | 5.3 | |
| | | | | |
| Chronically-infected monkeys (SIVmac251 for ~38 weeks) | | | | |
| RM ID | Gender | Age (years) | Necropsy weight (kg) | |
| 11-40 | Male | 2 | 6.4 | |
| 11-150 | Male | 2 | 5.7 | |
| 10-138 | Male | 3 | 6.7 | |
| 10-68 | Male | 3 | 6.3 | |
| 10-75 | Female | 3 | 6.5 | |
| 4-203 | Female | 9 | 7.8 | |
| 10-111 | Female | 3 | 5.9 | |
| 10-189 | Female | 3 | 4.9 | |
| | | | | |
| Uninfected Healthy monkeys | | | | |
| RM ID | Gender | Age (years) | Necropsy weight (kg) | |
| DE8M | Female | 5 | 6.2 | |
| M280 | Female | 4 | 5.1 | |
| 4-63 | Female | 10 | 5.0 | |
| H375 | Female | 12 | 6.8 | |
| J483 | Female | 20 | 6.5 | |
| J430 | Male | 21 | 7.7 | |
| H704 | Male | 13 | 8.0 | |
| | | | | |
| Uninfected monkeys with Chronic Enterocolitis (CE) | | | | |
| RM ID | Gender | Age (years) | Necropsy weight (kg) | CE duration (months) |
| 9-172 | Male | 6 | 10.1 | 30 |
| 13-055 | Female | 2 | 2.3 | 21 |
| 4-167 | Female | 11 | 4.5 | 7 |
