# Supplementary material for: Infectious SIV resides in adipose tissue and induces metabolic defects in chronically infected rhesus macaques
Source: Retrovirology. 2016 Apr 27;13:30. doi: 10.1186/s12977-016-0260-2 (PMC4847269; doi:10.1186/s12977-016-0260-2)
Supplement: Supplementary file 6 — 10.1186/s12977-016-0260-2 Primers used for nested PCR detection of SHIV-SF162p3 Gag and Env genes in AT-SVF cells of acutely infected rhesus macaques. [file 12977_2016_260_MOESM6_ESM.ppt]

## Slide 1
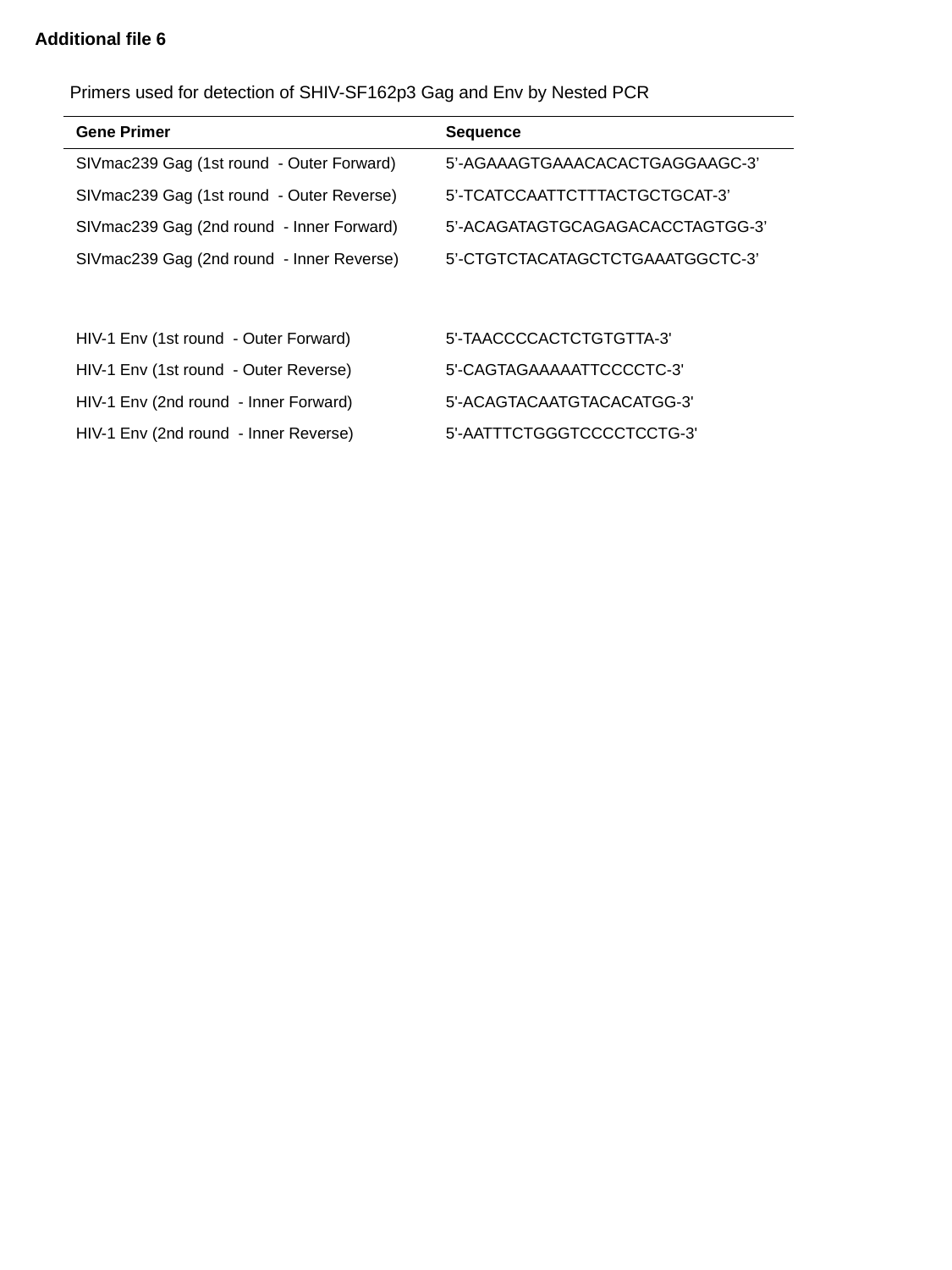

Additional file 6
Primers used for detection of SHIV-SF162p3 Gag and Env by Nested PCR
| Gene Primer | Sequence |
| --- | --- |
| SIVmac239 Gag (1st round - Outer Forward) | 5’-AGAAAGTGAAACACACTGAGGAAGC-3’ |
| SIVmac239 Gag (1st round - Outer Reverse) | 5’-TCATCCAATTCTTTACTGCTGCAT-3’ |
| SIVmac239 Gag (2nd round - Inner Forward) | 5’-ACAGATAGTGCAGAGACACCTAGTGG-3’ |
| SIVmac239 Gag (2nd round - Inner Reverse) | 5’-CTGTCTACATAGCTCTGAAATGGCTC-3’ |
| | |
| HIV-1 Env (1st round - Outer Forward) | 5'-TAACCCCACTCTGTGTTA-3' |
| HIV-1 Env (1st round - Outer Reverse) | 5'-CAGTAGAAAAATTCCCCTC-3' |
| HIV-1 Env (2nd round - Inner Forward) | 5'-ACAGTACAATGTACACATGG-3' |
| HIV-1 Env (2nd round - Inner Reverse) | 5'-AATTTCTGGGTCCCCTCCTG-3' |
