# Supplementary material for: Infectious SIV resides in adipose tissue and induces metabolic defects in chronically infected rhesus macaques
Source: Retrovirology. 2016 Apr 27;13:30. doi: 10.1186/s12977-016-0260-2 (PMC4847269; doi:10.1186/s12977-016-0260-2)
Supplement: Supplementary file 7 — 10.1186/s12977-016-0260-2 Primers used for SYBR Green real-time PCR analyses of mature adipocytes (floater fraction) of rhesus macaques. [file 12977_2016_260_MOESM7_ESM.ppt]

## Slide 1
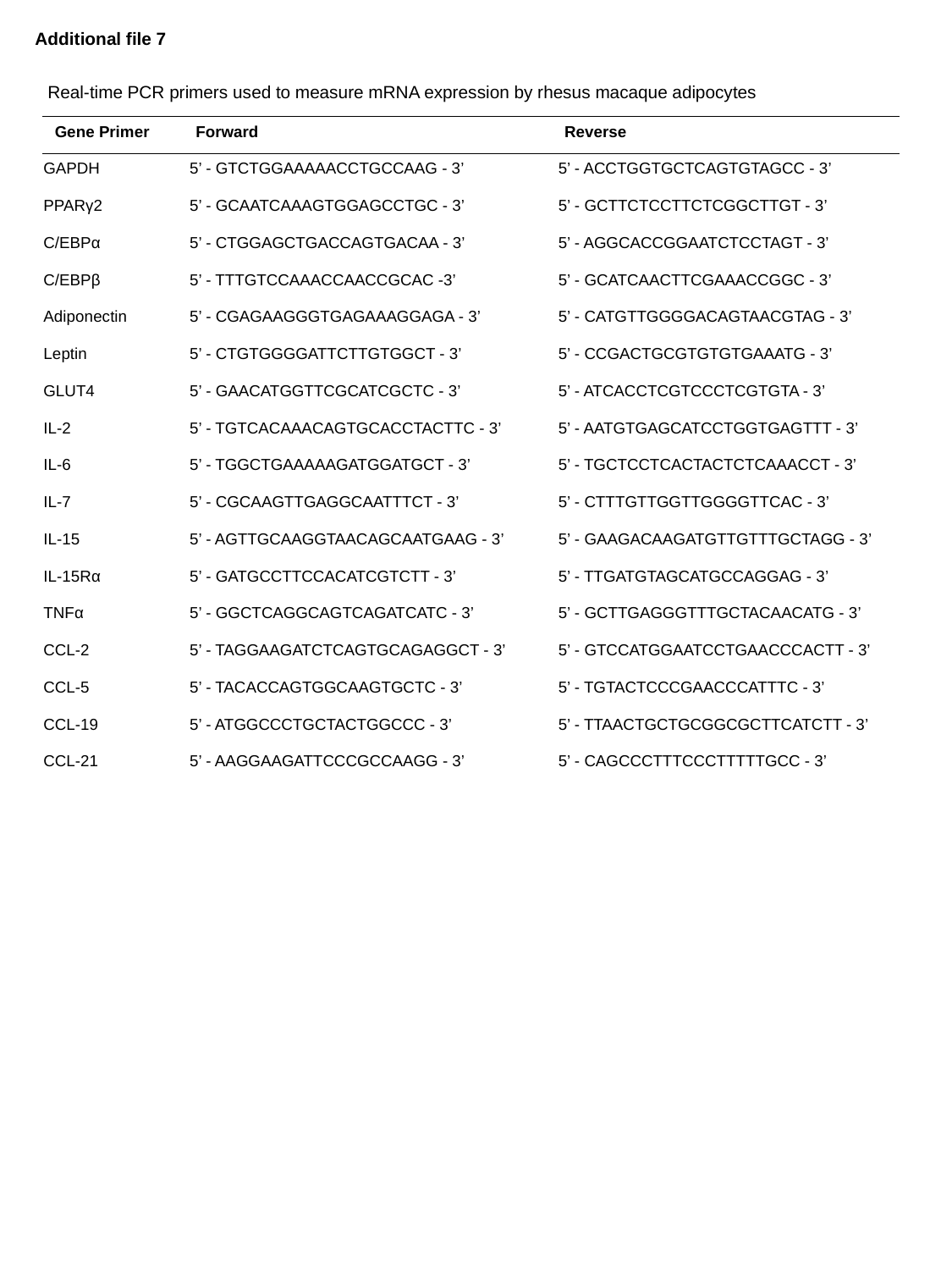

Additional file 7
Real-time PCR primers used to measure mRNA expression by rhesus macaque adipocytes
| Gene Primer | Forward | Reverse |
| --- | --- | --- |
| GAPDH | 5’ - GTCTGGAAAAACCTGCCAAG - 3’ | 5’ - ACCTGGTGCTCAGTGTAGCC - 3’ |
| PPARγ2 | 5’ - GCAATCAAAGTGGAGCCTGC - 3’ | 5’ - GCTTCTCCTTCTCGGCTTGT - 3’ |
| C/EBPα | 5’ - CTGGAGCTGACCAGTGACAA - 3’ | 5’ - AGGCACCGGAATCTCCTAGT - 3’ |
| C/EBPβ | 5’ - TTTGTCCAAACCAACCGCAC -3’ | 5’ - GCATCAACTTCGAAACCGGC - 3’ |
| Adiponectin | 5’ - CGAGAAGGGTGAGAAAGGAGA - 3’ | 5’ - CATGTTGGGGACAGTAACGTAG - 3’ |
| Leptin | 5’ - CTGTGGGGATTCTTGTGGCT - 3’ | 5’ - CCGACTGCGTGTGTGAAATG - 3’ |
| GLUT4 | 5’ - GAACATGGTTCGCATCGCTC - 3’ | 5’ - ATCACCTCGTCCCTCGTGTA - 3’ |
| IL-2 | 5’ - TGTCACAAACAGTGCACCTACTTC - 3’ | 5’ - AATGTGAGCATCCTGGTGAGTTT - 3’ |
| IL-6 | 5’ - TGGCTGAAAAAGATGGATGCT - 3’ | 5’ - TGCTCCTCACTACTCTCAAACCT - 3’ |
| IL-7 | 5’ - CGCAAGTTGAGGCAATTTCT - 3’ | 5’ - CTTTGTTGGTTGGGGTTCAC - 3’ |
| IL-15 | 5’ - AGTTGCAAGGTAACAGCAATGAAG - 3’ | 5’ - GAAGACAAGATGTTGTTTGCTAGG - 3’ |
| IL-15Rα | 5’ - GATGCCTTCCACATCGTCTT - 3’ | 5’ - TTGATGTAGCATGCCAGGAG - 3’ |
| TNFα | 5’ - GGCTCAGGCAGTCAGATCATC - 3’ | 5’ - GCTTGAGGGTTTGCTACAACATG - 3’ |
| CCL-2 | 5’ - TAGGAAGATCTCAGTGCAGAGGCT - 3’ | 5’ - GTCCATGGAATCCTGAACCCACTT - 3’ |
| CCL-5 | 5’ - TACACCAGTGGCAAGTGCTC - 3’ | 5’ - TGTACTCCCGAACCCATTTC - 3’ |
| CCL-19 | 5’ - ATGGCCCTGCTACTGGCCC - 3’ | 5’ - TTAACTGCTGCGGCGCTTCATCTT - 3’ |
| CCL-21 | 5’ - AAGGAAGATTCCCGCCAAGG - 3’ | 5’ - CAGCCCTTTCCCTTTTTGCC - 3’ |
